# New Insight in the Occurrence of Early Blight Disease on Potato Reveals High Distribution of *Alternaria solani* and *Alternaria protenta* in Serbia

**DOI:** 10.3389/fmicb.2022.856898

**Published:** 2022-03-23

**Authors:** Žarko Ivanović, Jovana Blagojević, Gordana Jovanović, Borko Ivanović, Danica Žeželj

**Affiliations:** ^1^Department of Plant Disease, Institute for Plant Protection and Environment, Belgrade, Serbia; ^2^Agricultural Advisory Service, Leskovac, Serbia; ^3^Agricultural Advisory Service, Čačak, Serbia

**Keywords:** *Alternaria solani*, *A. protenta*, *A. linariae*, *A. grandis*, Solanaceae

## Abstract

Early blight is an economically important disease of potato worldwide. Understanding which fungal pathogens are the causal agents of early blight and their distribution on the same host is essential to finding the best strategy for the control of this disease. Previous studies have shown that *Alternaria solani* is the main early blight pathogen parasitizing potato. Here, we analyzed genetic and phenotypic diversity in isolates of *Alternaria* spp. covering all potato production areas in Serbia. We showed that the four species of *Alternaria* were found in areas with different distributions of the species. The occurrence of *Alternaria* spp. was studied by analyzing isolates from symptomatic potato leaves during multiyear sampling. In addition to *Alternaria solani,* we detected three more large-spored species identified as *A. linariae* (syn. *A. tomatophila*), *A. protenta,* and *A. grandis* that were involved in early blight disease on naturally infected potato leaves in Serbia. Differentiation of species was supported by phylogeny obtained from the DNA sequences of the *GAPDH*, *calmodulin* and *Rpb2* genes. Our findings present a new perspective into the population structure of large-spored *Alternaria* species associated with early blight disease. Within the groups of large-spored *Alternaria* present in Serbia, evidence of *A. protenta* at high frequency reveals new insight into the contribution of *Alternaria* species in early blight disease. This work opens new perspectives for early blight management, while the distribution of different species on the same host suggests that the etiology of disease could depend on crop organization and the presence of other *Alternaria* hosts in close proximity to potato plants.

## Introduction

Early blight is one of the most important fungal diseases of potato and is spread worldwide in all major potato growing areas. Under favorable weather conditions, during warm and wet seasons, it can cause severe yield losses (Rotem, 1994; Batista et al., 2006). The pathogen spreads through infected potato tubers as well as through the various members of the Solanaceae family which may serve as reservoirs of primary inoculum (Rotem, 1994). Wind and periods of rain favor sporulation and could lead to disease outbreaks (Van der Waals et al., 2004). However, potato is severely affected by early blight every year, causing premature defoliation with a significant impact on tuber yield and potato quality loss of up to 50% annually (Shtienberg et al., 1990; Van der Waals et al., 2004; Leiminger et al., 2014; Landschoot et al., 2017). In Serbia, potato is produced as a field crop in several regions of the country. In 2020, potato was grown on 29,676 ha with a production of 664,891 tons (FAOSTAT, 2022). In all potato production areas, weather conditions are conducive to the development of early blight disease, and epidemics can occur at any phase of potato growth and is more severe during the late phase of the growth.

Four *Alternaria* species with large conidia have been reported as causal agents of early blight on potato: *A. solani, A. grandis, A. protenta,* and *A. linariae* (syn. *A. tomatophila*; Simmons, 2000; Gannibal et al., 2014; Landschoot et al., 2017). *A. solani* is the primary species causing early blight of potato worldwide, whereas other three species were reported sporadically. *A. grandis* on potato is reported in several different countries: Algeria (Bessadat et al., 2017), Brazil (Rodrigues et al., 2010), Belgium (Landschoot et al., 2017), and the United States (Simmons, 2000). *A. linariae* (syn. *A. tomatophila*) is known as an important pathogen causing early blight of tomato (Gannibal and Orina, 2013), although it has also been detected in potato (Ayad et al., 2018). *A. protenta* was first described as a pathogen of *Helianthus annuus* (Simmons, 1997) but has recently been identified as a member of the *Alternaria* genus that also causes early blight in potatoes (Woudenberg et al., 2014; Landschoot et al., 2017; Ayad et al., 2018), although it is economic impact and significance as a potato pathogen remains to be evaluated.

Identification and characterization of *Alternaria* spp. populations are essential for understanding the etiology and epidemiology of early blight and for the improvement of disease control strategies. Molecular markers routinely used for the identification and phylogenetic analysis of early blight-causing agents are the *ITS* region (internal transcribed spacers 1 and 2 and intervening 5.8S rDNA gene; White et al., 1990), glyceraldehyde-3-phosphate dehydrogenase (*GAPDH*) gene (Berbee et al., 1999), RNA polymerase II gene (*Rpb2*; Liu et al., 1999), translation elongation Factor 1-alpha (*TEF 1α*; O’Donnell et al., 1998; Carbone and Kohn, 1999), *Alternaria* major allergen gene *Alt a 1* (Hong et al., 2005), and anonymous gene region (*OPA10-2*; Andrew et al., 2009).

Since there is very little knowledge on early blight disease in Serbia, the aim of this study was to gain insight into the distribution of large-spored *Alternaria* spp. present on potato leaves. Therefore, the main objectives for the current study were to: (i) study the occurrence and distribution of early blight disease on potato in Serbia, (ii) identify *Alternaria* spp. causing agents of early blight on potato in the main production areas of Serbia; (iii) to determine the genetic relationship of Serbian *Alternaria* spp. isolates with strains identified in different parts of the world.

## Materials and Methods

### Survey, Sample Collection, and Fungal Isolation

To identify the presence and distribution of *Alternaria* spp., a 3-year survey was conducted from 2016 to 2018. Potato plants with early blight symptoms were collected from large commercial fields and from a small farmer’s garden. Samples were collected in nine different districts of Serbia: North Bačka, South Bačka, Pomoravlje, Moravica, Raška, Jablanica, Zlatibor, Rasina, and City of Belgrade district. Sixty-one fields in 20 different localities were surveyed (Table 1). Infected plants were sampled at the production plots in two diagonal transects, and two leaves per plant were collected from 10 plants at equal distances across a diagonal transect of the field during early blight disease epidemics between July and September. Samples with dark and circular lesions with typical concentric rings were collected. For *Alternaria* isolation, necrotic leaf cuts from a margin of the lesion were surface disinfected in 1% sodium hypochlorite, rinsed with sterile distilled water, transferred to Petri dishes containing potato dextrose agar (PDA, Difco, Detroit, MI), and incubated at 23°C. The morphological characteristics of the isolates were studied by taking a 5 mm mycelial plug from the growing colony margin of a 5-day-old culture and subculturing on V8 medium (200 ml V8 juice Campbell; 3 g CaCO_3_; 15 g agar; and 800 ml of sterile distilled water). The cultures were placed under cool white fluorescent light with 12 h/12 h periods of light/dark and incubated on 23°C for 5 days (Simmons, 2007).

**Table 1:** Localities and number of fields where *Alternaria* spp. were collected.

Districts	Localities	Number of fields	Number of isolates	Potato cultivars
North Bačka	Maglić	7	58	Lady Claire, VR808
	Kulpin	4	10	Lady Claire
South Bačka	Zobnatica	4	8	VR808, Pirol
Pomoravlje	Dragocvet	3	16	Marabel
	Međureč	3	16	Liseta
Moravica	Zablaće	2	4	Arizona, Bellarosa
	Baluga	4	10	Opal, Sinora
	Veles	5	26	Arizona, Rudolf, Agria
	Krivača	1	6	Aladin
	Katići	3	6	Agria
Raška	Rudno	2	2	Agria
	Bzovik	3	4	Carrera, Memphis
Jablanica	Bogojevce	6	32	Arizona
	Navalin	3	8	Arizona
	Brejanovce	2	6	Arizona
	Pečenjevce	2	8	Arizona
Zlatibor	Kladnica	2	4	Agria
	Drmanovići	1	2	Agria
Rasina	Velika Drenova	2	2	Agria
City of Belgrade	Borča	2	2	Kenebek

### Morphological Identification

The growing cultures of all isolates were studied with a protocol described by Simmons (2007). Conidial morphology was examined using a microscope (Olympus BX51TF, Japan) at 400 × magnification. Dimensions are based on the observation of 100 conidia and 50 conidiophores per isolate. The conidia length and sporulation pattern data were analyzed using the analysis of variance (ANOVA; *p* < 0.05) in the statistical software package SPSS version 20.0 (SPSS Inc., Chicago, IL, United States).

### Molecular Identification

For molecular identification, 30 mg of dry weight mycelium was collected from PDA plates and used for DNA extraction according to the manufacturer’s instructions of the DNeasy Plant Mini Kit (Qiagen, Valencia, CA, United States). Polymerase chain reaction (PCR) was used with the specific primers OAsF7/OAsR6 which amplified a 164 bp fragment from the *Alt a1* gene to separate the species *A. solani* and related species (*A. grandis* and *A. protenta*) from *A. linariae,* and with the primer pair OAtF4/OAtR2, which amplified a 483 bp fragment from the calmodulin-encoding gene of *A. linariae* (Gannibal et al., 2014; Supplementary Table 1). PCR was performed in a Mastercycler Nexus GSX1 (Eppendorf, Hamburg, Germany) with the amplification program as described in Gannibal et al. (2014). The PCR amplicons were separated by electrophoresis on 1% agarose gels run in 1 × TBE buffer at 90 V constant voltages. The gels were stained with ethidium bromide and visualized under UV light. To differentiate between *A. grandis* and *A. solani* (including *A. protenta*), PCR identifications of isolates that amplified a 164 bp fragment with the primer pair OasF7/OasR6 were complemented by RFLP of the *calmodulin* gene that was amplified using the primer pair CALDF1/CALDR1 (Lawrence et al., 2013). The resulting PCR products were cut using double restriction with enzymes *HaeII* and *RsaI* (New England Biolabs) according to Ayad et al., (2017a). RFLP patterns were discriminated by the size of the larger fragment, 420 bp for *A. solani* and 292 bp for *A. grandis*. To distinguish between *A. solani* and *A. protenta* isolates, sequencing of the *Rpb2* locus was performed.

Multilocus sequence analysis was performed for all 230 isolates with amplification of three housekeeping genes by primers RPB2DF/RPB2DR (Lawrence et al., 2013) for the (*Rpb2*) gene, primer pair CALDF1/CALDR1 for the amplification of *Calmodulin* gene (Lawrence et al., 2013), and by using GPD1/GPD2 primers for the amplification of (*GAPDH*; Berbee et al., 1999; Supplementary Table 1). The PCR amplification conditions for *RPB2* and *calmodulin* genes were described in Lawrence et al., (2013), whereas *GAPDH* gene amplification was conducted using the PCR conditions described in Berbee et al., (1999). The PCRs were conducted in a Mastercycler Nexus GSX1 (Eppendorf, Hamburg, Germany) in a 25 μl reaction mixture using the following final concentrations or total amounts: 5 ng DNA, 1 × PCR buffer (20 mM Tris/HCl pH 8.4 and 50 mM KCl), 1 μM of each primer, 2.5 mM MgCl_2_, 0.25 mM of each dNTP, and 1 unit of Taq polymerase (Fermentas, Vilnus, Lithuania). The amplified PCR products were separated by electrophoresis and purified (QIAquick PCR Purification Kit, Qiagen, Valencia, CA, United States) for Sanger sequencing in both directions (Macrogene, Seoul, Korea). For final identification, the DNA sequences of all isolates were compared with reference sequences of *Alternaria* species using the BLAST program of NCBI. From DNA sequences of 230 isolates, 37 were selected based on the species and geographic location and as representative strains these sequences were deposited into NCBI GenBank (Supplementary Table 2).

### Construction of Phylogenetic Tree

Partial gene sequences of *GAPDH* (569 bp), *Rpb2* (772 bp), and *calmodulin* (778 bp) were concatenated (2,119 bp) to conduct a multilocus phylogenetic tree. Analysis is done after the gene sequences are concatenated head to tail to form a gene alignment which is then analyzed to generate the species tree. Manual corrections, alignments, and comparisons of the aligned database were performed using CLUSTALW integrated into MEGA version 11.0 software (Tamura et al., 2021). The gamma distributed Tamura-Nei model determined by ModelTest implemented in MEGA11 was used as the best fitting model of nucleotide substitution. The reliability of the obtained trees was evaluated using 1,000 bootstrap replicates and bootstrap confidence values <50% were omitted. Phylogenetic trees were constructed using the maximum likelihood (ML) method implemented in the same software.

The phylogenetic relationships among *Alternaria* spp. from potato were confirmed by Bayesian phylogeny. For concatenated data, the Bayesian information criterion (BIC) was conducted using the best fitting substitution model according to jModeltest 2.1.7 (Darriba et al., 2012). The Bayesian analysis was performed in MrBayes 3.1.2 (Huelsenbeck and Ronquist, 2003) for 1 million generations, sampling every 100 trees, using simultaneous Markov chain Monte Carlo (MCMC) runs. The standard deviation of the split frequencies was checked until it reached a value below 0.01 (default burnin = 25%). The convergence of the MCMC chains and their stationarity were confirmed using Tracer 1.5 (Rambaut and Drummond, 2009). The phylogenetic tree was observed and printed using FigTree 1.4 (Rambaut and Drummond, 2012).

### Pathogenicity Test

Pathogenicity tests were conducted with all investigated isolates by inoculating leaves of potato plants (Carrera variety) in pot experiments. Potato seed tubers were sown in pots filled with sterilized soil in a glass house at 25°C with an 8 h/16 h photoperiod. One month old plants were used for inoculations. The conidial suspensions prepared from cultures grown on V8 agar at 23°C for 5 days were adjusted to 1 × 10^6^ conidia/ml, and a 20 μl drop was applied to the leaf surface. Control plants were treated at the same time using sterile distilled water. After inoculation, plants were placed in a growing chamber with relative humidity between 95 and 100% for one week at 23°C. The test was repeated three times. Fragments of lesion areas were cut and surface sterilized before being placed into V8 agar. The morphological characteristics of reisolated pathogens were compared with original isolates to confirm Koch’s postulates. Inoculated leaves were cut from the tested potato plants, and the necrotic leaf area was measured using the program ImageJ (Schneider et al., 2012). In the pathogenicity test, the Kruskal–Wallis test was used in the statistical software package SPSS version 20.0 (SPSS Inc., Chicago, IL, United States).

## Results

### Presence and Distribution

**Figure 1:**
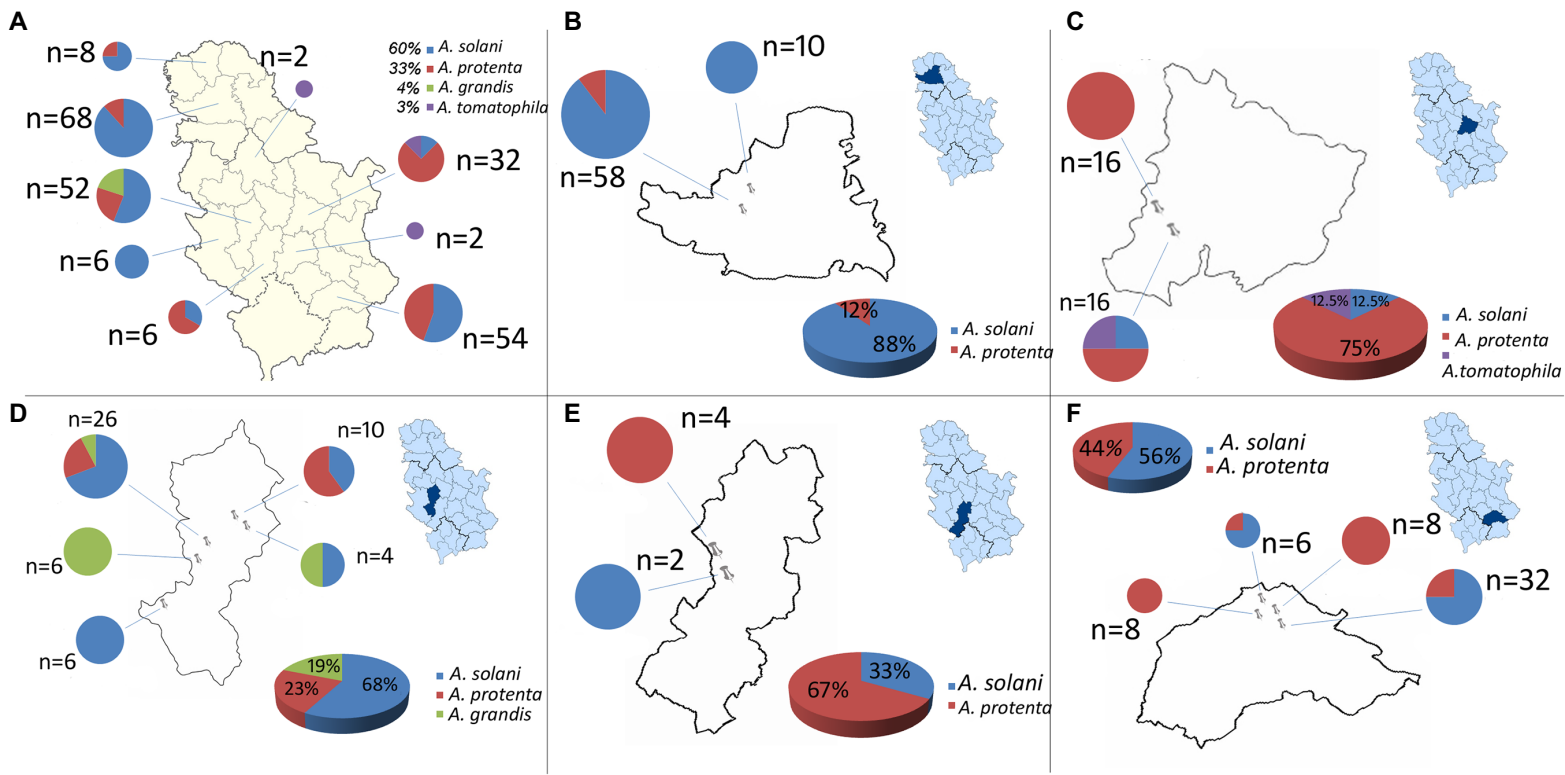
Geographical distribution of large-spored *Alternaria* spp.: **(A)** Serbia, **(B)** South Bačka district (localities: Maglić, Kulpin), **(C)** Pomoravlje district (localities: Međureč, Dragocvet), **(D)** Moravica district (localities: Veles, Krivača, Katići, Baluga, Zablaće), **(E)** Raška district (localities: Bzovik, Rudno), and **(F)** Jablanica district (localities: Pečenjevce, Brejanovce, Navalin, Bogojevce).

**Figure 2:**
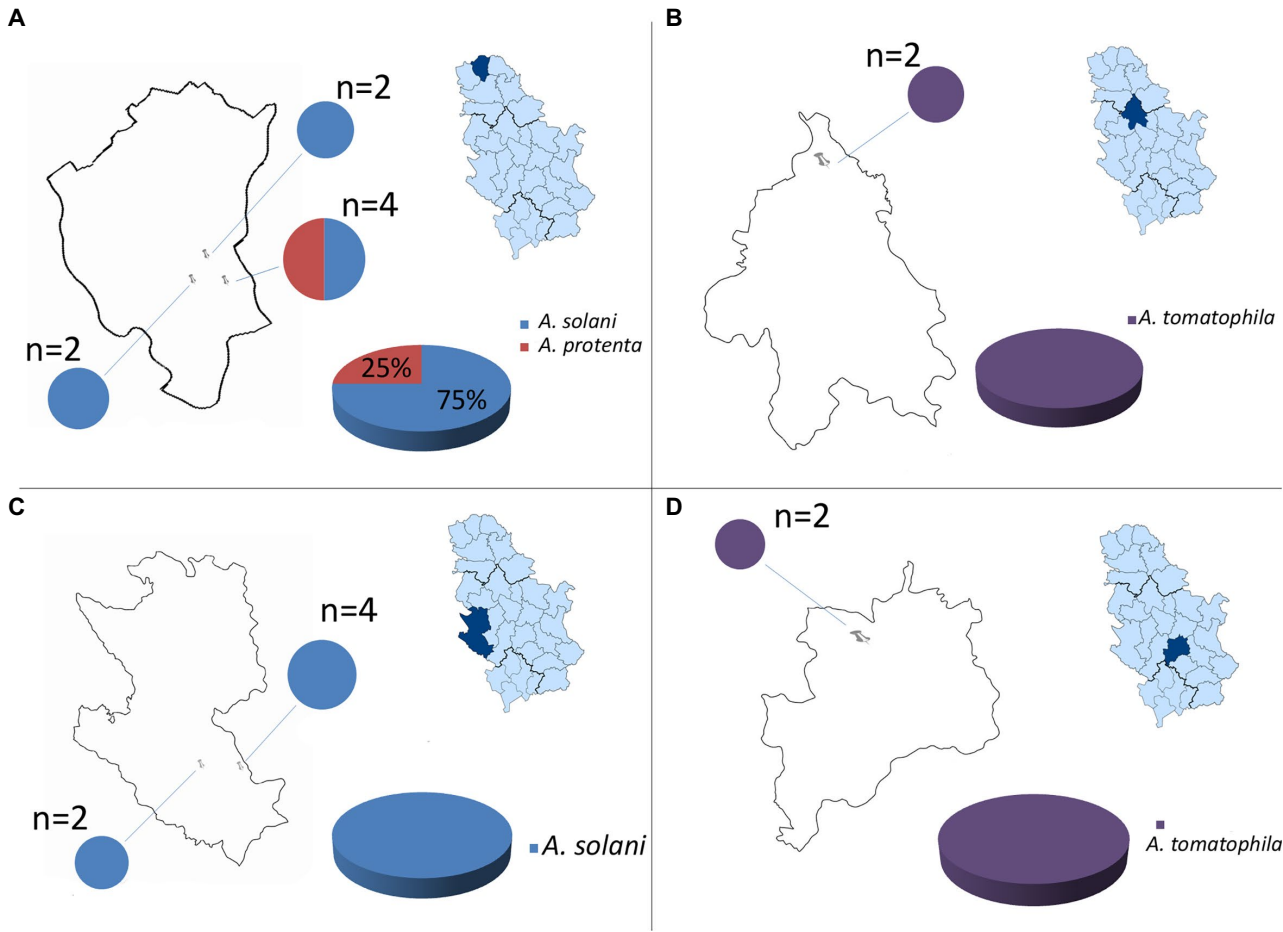
Geographical distribution of large-spored *Alternaria* spp.: **(A)** North Bačka district (locality: Zobnatica), **(B)** Belgrade district (locality: Borča), **(C)** Zlatibor district (localities: Drmanovići, Kladnica), and **(D)** Rasina district (locality: Velika Drenova).

Throughout a three-year survey (2016–2018), the presence of early blight was confirmed in all surveyed localities and four large-spored *Alternaria* pathogens: *A. solani, A. protenta, A. grandis,* and *A. linariae* were detected on potatoes in Serbia (Figures 1, 2). In total, 230 *Alternaria* spp. isolates were recovered from symptomatic potato leaves collected in 40 different potato fields (Table 1). Most of the isolated pathogens belonged to *A. solani*, accounting for 60% of the overall isolates. *A. solani* was not recorded in five localities: Borča (district City of Belgrade), Velika Drenova (district Rasina), Bzovik (district Raška) Navalin, and Pržine (district Jablanica). *A. protenta* was detected in 33% of the isolates in all major potato production areas, except in the localities Velika Drenova (district Rasina), Rudno (district Raška), Pečenjevce (district Jablanica), Krivača, Zablaće, Katići (district Moravica), Drmanovići, and Blato (district Zlatibor). *A. grandis* and *A. linariae* were identified in 4 and 3%, respectively. *A. grandis* was found in Krivača and Zablaće (district Moravica), while *A. linariae* was detected in Velika Drenova (district Rasina), Dragocvet (district Pomoravlje), and Borča (district City of Belgrade).

### Morphological and Molecular Identification of *Alternaria* Spp.

Morphological preliminary identification of fungi was performed 7 days after incubation on V-8 medium, and large-spored *Alternaria* isolates revealed slight variability in colony morphology. According to Simmons (2007), isolated species were identified by their spore size, and the isolates could be separated into four species: *A. solani, A. protenta, A. grandis,* and *A. linariae.*

*A. solani* isolates produced colonies with conidia dimensions as follows: 190 ± 24 μm in length and 16 ± 2 μm in width with 5–13 transverse and up to three longitudinal septa and branching conidiophores 187.7 ± 4.8 μm with numerous conidiogenous sites and spores. Isolates of *A. protenta* developed conidia 225 ± 23 μm in length and 17 ± 2 μm in width with 8–13 transverse septa and one longitudinal septum in a few transverse segments. Conidiophores were solitary, reaching 83 μm in primary growth up to 210 μm in the stage of secondary branching (137.7 ± 6.9 μm). *A. linariae* isolates had solitary conidiophores up to 142 μm long (133.7 ± 7.5 μm), rising straight from the surface with large conidia. The size of the spores was 255 ± 33 μm in length and 18 ± 2 μm in width with 1–12 transverse septa and most commonly only one longitudinal septum in many transverse segments. Isolates identified as *A. grandis* produced conidia 262 ± 36 μm in length and 16 ± 3 μm in width with 7–12 transverse septa and one longitudinal septum in a few transverse segments. Conidiophores arose as singly, up to 200 μm in length (157.2 ± 9.3 μm).

Assumptions for conducting statistical analyses were inspected using Shapiro–Wilk’s normality test, Box’s test of equality of covariance matrices and Levene’s test of equality of error variances. Data showed adequate values for conducting parameter statistical test one-way ANOVA for conidiophores and conidial length but not for conidial width so this category was excluded from the further statistical analyses. A significant difference among the identified groups was found only for conidial length *p* = 0.002. Parameters for conidial length indicated again that *A. solani* and *A. protenta* did not show significant differences in conidial size (*p* = 0.122).

To confirm morphological identification of *Alternaria* isolates, the primer pair OAsF7/OAsR6 amplified the DNA fragment at position 164 bp for 222 isolates (Supplementary Figure 1A). The second primer pair OAtF4/OAtR2 was used to confirm that *A. tomatophila* amplified a 483 bp fragment for eight isolates (Supplementary Figure 1B). For species differentiation between *A. solani/A.protenta* and *A. grandis* isolates, RFLP was performed with *HaeII* and *RsaI* restriction enzyme digestion of PCR products of the *calmodulin* gene. The restriction pattern of the *A. solani/A.protenta* isolates exhibited a larger fragment at 420 bp; in contrast, the restriction pattern of the *A. grandis* isolates exhibited a larger fragment at 292 bp (Supplementary Figure 1C). Additionally, sequencing of the *Rpb2* gene was used to differentiate *A. solani* and *A. protenta* isolates. BLAST analyses of the *Rpb2* gene separated *A. protenta* from *A. solani isolates* and showed that *A. protenta* isolates had 100% nucleotide sequence identity with the corresponding gene regions of *A. protenta* strain CBS 116696. Molecular identification resulted in 138 *A. solani* isolates, 74 *A. protenta* isolates, 10 *A. grandis* isolates, and 8 *A. linariae* isolates collected from potato in Serbia. After sequences analysis of all 230 isolates, DNA sequences of the *GAPDH*, *calmodulin*, and *Rpb2* genes were identical for the same species and we selected 37 isolates based on species and geographic location to be representative for phylogenetic analysis.

### Molecular Characterization

**Figure 3:**
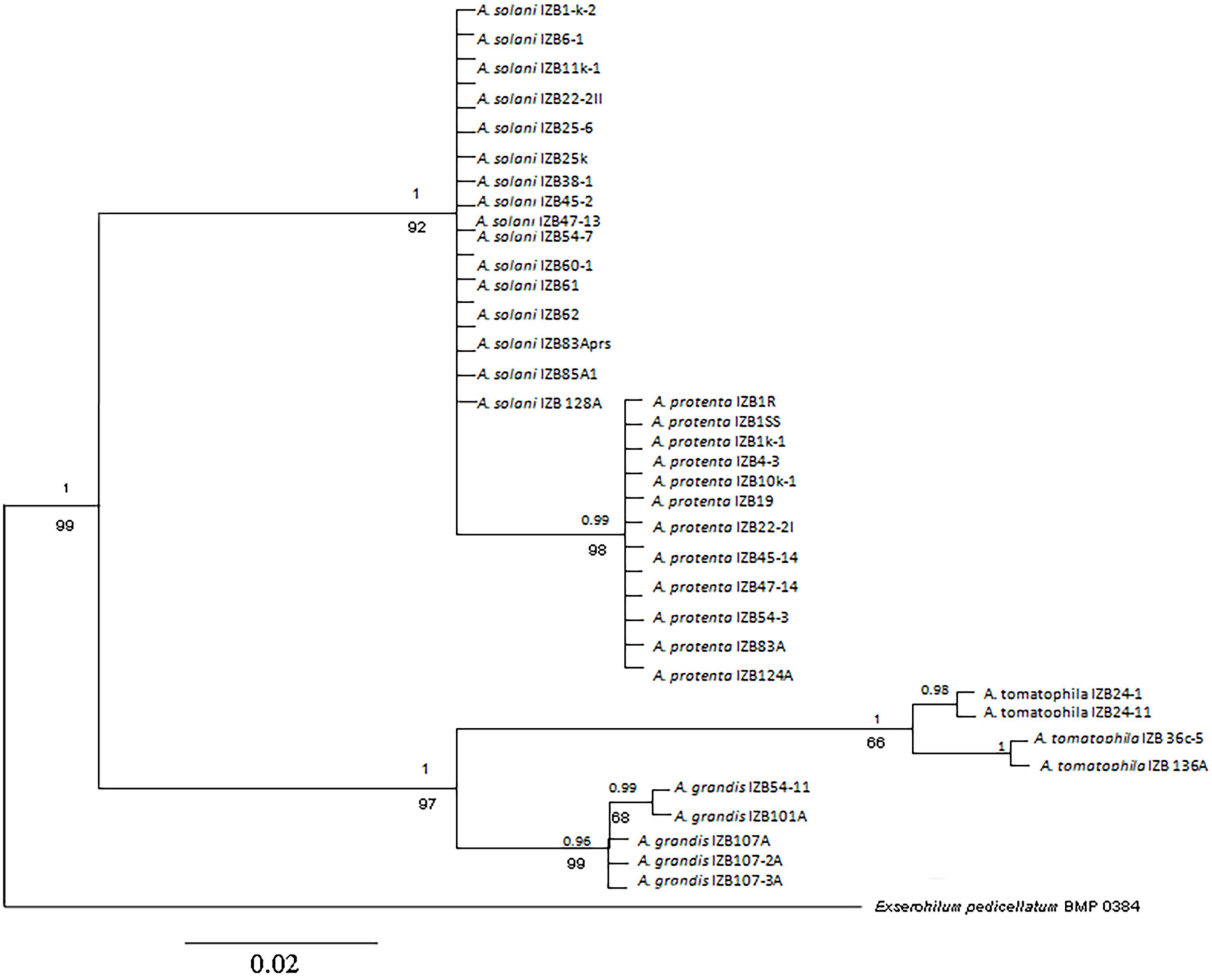
Phylogenetic tree based on Bayesian analysis of the concatenated sequences of the *GAPDH, Rpb2,* and *calmodulin* genes of *Alternaria* strains from potato. Bayesian posterior probabilities are shown above branches, and maximum likelihood bootstrap support values are indicated below branches. Bar: the estimated nucleotide substitutions per site are 0.02. The numbers near each branch represent percentages out of 1,000 bootstrap replications.

Multilocus sequence analysis of each individual sequence of the *GAPDH, calmodulin,* and *Rpb2* genes and comparing all investigated isolates with reference GenBank sequences (*A. linariae* CBS 109156, *A. protenta* CBS 116696, *A. solani* CBS 109157, and *A. grandis* CBS 109158), identified four different species distributed in four clusters (Figure 3; Supplementary Figures 2–4). Phylogenetic trees constructed for *GAPDH* and *calmodulin* genes were similar and grouped isolates into three diverse clusters that identified isolates as *A. solani/A. protenta, A. grandis*, and *A. linariae*, while phylogenetic trees constructed for the *Rpb2* gene followed the results of previous identification and identified four different species.

PCR products of the *calmodulin* regions revealed two clades for *A. grandis* and *A. linariae*. The first cluster, *A. linariae,* separated as a monophyletic lineage with a bootstrap value of 96%, segregated four isolates into two subclusters along with the reference strains. One clade grouped isolates IZB 136A and IZB 36c-5 with reference strains CBS 109156, MF-P138061, and DA 100 obtained from the NCBI database, and the second clade grouped isolates IZB 24–1 and IZB 24–11 with reference strain DA 119. The cluster that included *A. grandis* isolates was divided into two subclusters, one with two isolates, IZB 101A and IZB 54–11, and the reference strains NB 251 and DA 009. The second subcluster consisted of isolates IZB 107A, IZB 107-2A, and IZB 107-3A and reference strains DA 052 and CBS 109158.

Concatenated gene sequences of the *GAPDH, calmodulin,* and *Rpb2* genes were used to substantiate the results of separate gene analysis. Phylogenetic analyses by the Bayesian information criterion (BIC) employed in jModelTest confessed a general time reversible model with gamma distribution (TIM3 + IG) as the most suitable substitution model. The distance from the end to the end of the concatenated sequences used in this testing was 2,135 bp. Concatenated sequences of three analyzed gene regions, *GAPDH, calmodulin,* and *Rpb2*, in supplementary common phylogeny analysis were recognized as a single multilocus dataset for all isolated species. The parameters for the Bayesian analyses were Lset base = (0.2378 0.3131 0.2471) nst = 2 tratio = 1.3022 rates = equal pinvar = 0. For second phylogenetic analyses of *GAPDH, calmodulin,* and *Rpb2* concatenated sequences, maximum likelihood analysis was employed and the T92 model was selected as the best-fit model.

### Pathogenicity Test and Reisolation

**Figure 4:**
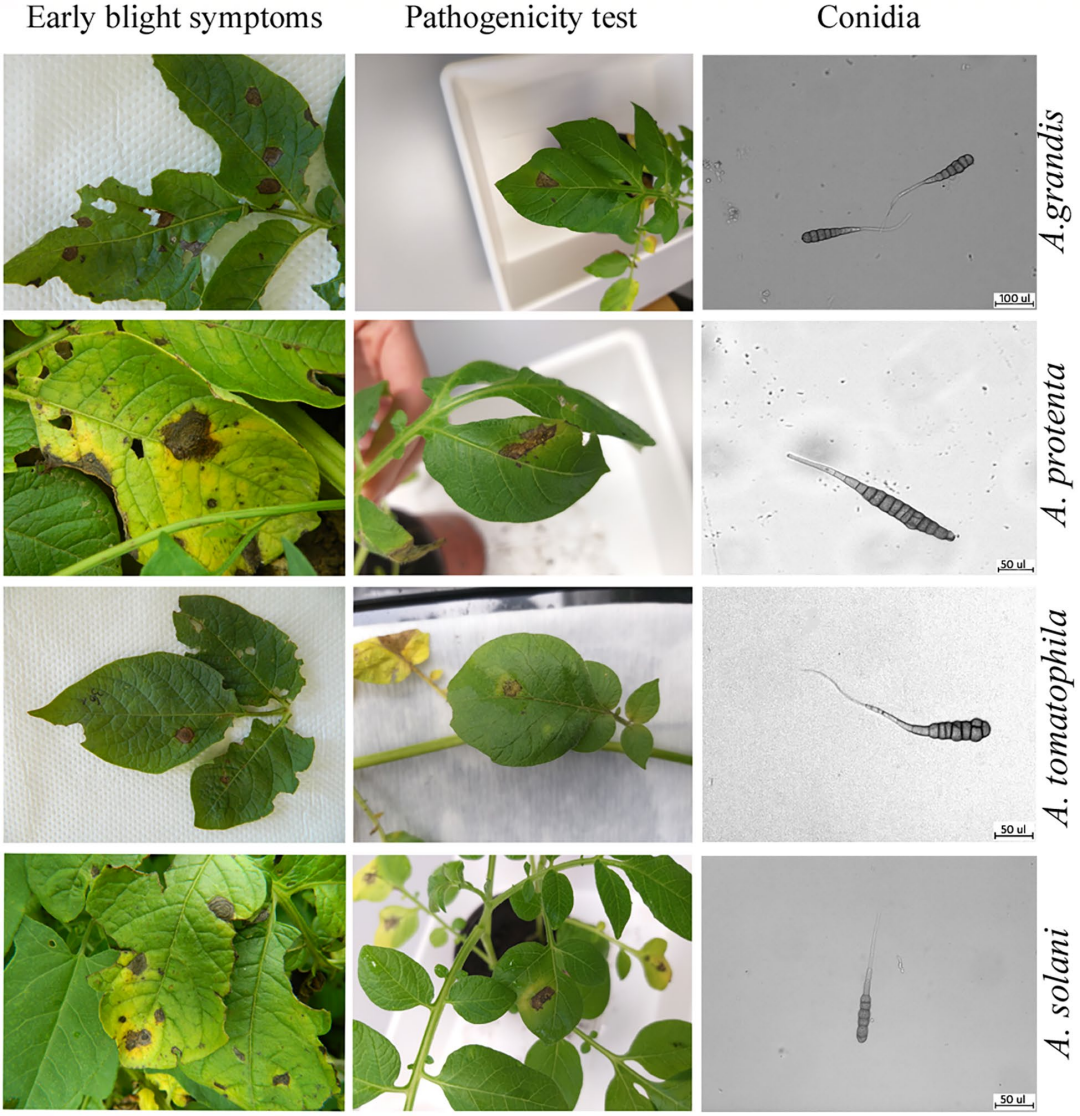
**(A)** Symptoms of early blight on potato; **(B)** pathogenicity test 7 days after inoculation **(C)** conidia.

After seven days of incubation, typical early blight disease symptoms were observed on inoculated potato plants for all tested isolates with differences in symptom appearance (Figure 4). To confirm Koch’s postulates, the pathogen was reisolated, and based on colonies and the morphology of conidia, recovered isolates were identical as to the original isolates.

During the first week after inoculation, the development of lesions was slow, and spots were small (≤ to 5 mm in diameter). In the second week after inoculation, symptoms appeared as brown to dark spots on leaves similar to those observed in the potato fields (Figure 4). Although inoculations with *A. solani*, *A. protenta*, *A. grandis*, and *A. linariae* isolates resulted in disease symptoms visible one week after inoculation, leaf spots differentiated in color and diameter. Concentric rings with dark color and small diameter were characteristic of isolates of *A. grandis* and *A. linariae*. *A. solani* was the most aggressive, with the largest spots and dark color, whereas *A. protenta* produced leaf spots light brown to dark in color. No symptoms were observed on plants inoculated with sterile distilled water.

**Figure 5:**
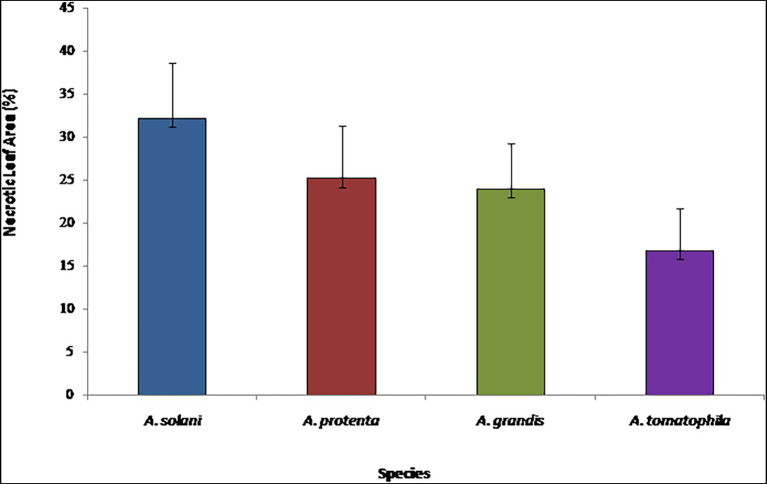
Virulence of *Alternaria solani, A. protenta, A. grandis,* and *A. linariae* on potato leaves, under controlled conditions 10 days after inoculation. Multiple-means comparisons were supported by Kruskal–Wallis test.

A Kruskal–Wallis test was conducted to determine if there were differences in aggressiveness between isolated *Alternaria* species on potato leaves. The distributions of necrotic leaf area (%) were not similar for all groups, as assessed by visual inspection of a boxplot. The necrotic areas of potato leaves were significantly different between the different species, χ^2^(3) = 39.142, *p* = 0.000. Subsequently, pairwise comparisons were performed using Dunn’s (1964) procedure with a Bonferroni correction for multiple comparisons. This post-hoc analysis revealed statistically significant differences in aggressiveness among *A. solani*, the most pathogenic species (32.1 ± 6.5), and *A. linariae,* the weakest pathogen (16.8 ± 4.8) compared to other species; however, *A. protenta* and *A. grandis* revealed moderate pathogenicity (25.2 ± 6.2 and 23.9 ± 5.3, respectively) with no statistically significant difference in pathogenicity on potato (*p* = 0.812; Figure 5).

## Discussion

In this study, we described the first results of a three-year survey on the presence and distribution of *Alternaria* spp. causing agents of early blight disease on potato in Serbia. The survey was conducted throughout nine growing regions of potato and showed the presence of four large-spored *Alternaria* species: *A. solani, A. protenta, A. grandis*, and *A. linariae,* which cause early blight disease of potato in Serbia. To the best of our knowledge, this is the first record of *A. protenta*, *A. grandis*, and *A. linariae* as causal agents of early blight of potato in Serbia. The presence of *A. solani* was found to be widely distributed in most of the surveyed potato fields but not the only fungus causing early blight. In most countries, *A. solani* is usually isolated as the main causative agent of early blight, as seen in Algeria (Bessadat et al., 2017; Ayad et al., 2017a), Germany (Hausladen, 2006; Leiminger et al., 2015), Brazil (Lourenco et al., 2009), Cuba (Martinez et al., 2004), China (Zhao et al., 2018), the United States (Ding et al., 2019; Adhikari et al., 2020), Russia (Gannibal et al., 2014), and South Africa (Van der Waals et al., 2004). Our research suggests that a different situation occurs with *Alternaria* spp. in Serbia, where *A. solani* was detected with an incidence of 60% in diseased potato plants. *A. protenta* was recently found to be a pathogen on potato (Landschoot et al., 2017; Ayad et al., 2017b), but overall, study of its presence and contribution to the population structure of *Alternaria* spp. were not performed thus far in Serbia. *A. protenta* was found to be distributed in the majority of the surveyed potato fields, and its occurrence indicates that this pathogen has been identified as an important pathogen among other *Alternaria* species causing early blight of potato in Serbia. *A. protenta* was recorded on potato in Algeria and Belgium but with a small contribution to the *Alternaria* population structure (Landschoot et al., 2017; Ayad et al., 2017a). The emergence of *A. protenta* at a high percent can imply that a shift in distribution species that cause early blight disease may be taking place but also could be that this specie being present in potato growing area for many years but stays undetected. Still we do not know where these species originate from and when they spread. This change could have a significant impact on disease management. *A. grandis* was detected only in one district with a relatively low frequency. This is in accordance with previous findings where *A. grandis* was observed on potato fields but was not the main causing agent of the disease (Rodrigues et al., 2010; Landschoot et al., 2017; Ayad et al., 2017a). This pathogen was found to produce black leaf spots smaller than other *Alternaria* species present on the plant with low severity of the disease. In some cases (Borča and Velika drenova), only *A. linariae*, as a large-spored *Alternaria*, was found on potato. *A. linariae,* the main causal agent of tomato early blight, was found in Serbia as a causative agent of the same disease on potato but only in the fields where tomato was grown next to potato. Interestingly, tomato served as a strong inoculum reservoir of *A. linariae,* which was able to infect potato when tomato is grown side by side potato. Potato fields in districts Jablanica and North Bačka, which were located only a few kilometers away from the main tomato production areas, were not affected by *A. linariae*.

BLAST analysis showed that sequences of 230 Serbian *Alternaria* spp. isolates could be separated into four groups, with all isolates clustered together with the reference isolates from different parts of the world. Sequences of Serbian *A. solani* isolates were identical to each other as well as to sequences of isolates from NCBI GenBank originating from Russia, Algeria, and the United States. *A. protenta* isolates from Serbia obtained in this study were grouped with NCBI GenBank sequences of isolates originating from Algeria, Israel, New Zealand, and Australia in phylogenetic analysis. Comparison of *calmodulin* sequences revealed two haplotypes among *A. linariae* isolates, and isolates derived from Dragocvet were similar to isolate originating from Algeria. The second haplotype sampled in Velika Drenova and Borča was similar to the sequences of the isolates originating from Russia, the United States, and Algeria. A maximum likelihood tree, constructed using the *calmodulin* sequences of *A. grandis* isolates, revealed the presence of two haplotypes grouped into two subclusters: one clade, isolates of *A. grandis* originating from Algeria and two Serbian *A. grandis* isolates derived from Zablaće and sampled in Veles. In the second clade, three Serbian isolates from Krivača were grouped together with isolates from Algeria and the United States.

Area of production in Serbia has declined during the last 10 years from 76,675 ha in 2010 to 29,676 ha in 2020 (FAOSTAT, 2022). At the same time, the composition of potato cultivars has changed, and imported cultivars have become prevalent. Domestic potato cultivars are very rare and can be found only in some house garden production. Based on sparse information regarding the status of *Alternaria* spp., it is impossible to fully conclude the etiology of these pathogens. For instance, if they were native in this area or they were imported *via* potato seed and established during the years with seed material of uncontrolled and uncertain health status. Additionally, the percentage of distribution of different large-spored *Alternaria* spp. that causes early blight varies in different potato growing regions; the presence of diverse *Alternaria* species on potato with the ascendency of *A. solani* is expected and in accordance with previous findings (Gannibal et al., 2014; Leiminger et al., 2015; Landschoot et al., 2017; Ayad et al., 2017a; Ding et al., 2019; Adhikari et al., 2020). Some fields, especially in South Bački and Zlatibor districts, showed dominancy of *A. solani* as the main causative agent of early blight disease. The composition of the *Alternaria* population in some observed fields, particularly in central and southern districts, showed a high percentage and, in some cases, total dominancy of *A. protenta*. The occurrence of *A. protenta* as the dominant early blight pathogen was not recorded before. The etiology of this population shift is still unknown and indicates a need for further investigation.

In the pathogenicity test, *A. solani* isolates represent the most virulent pathogenic species on potato, causing the largest leaf spots with premature defoliation, with a high negative impact on potato plant yield. The presence of other large-spored *Alternaria* species can be linked with their native host cultivated next to the potato field. In two potato fields surrounded by tomato plants, *A. linariae* was isolated as a main early blight. High inoculum of *A. linariae* from tomato that was grown over the years in the same place had prevalence on potato. Thus, *A. linariae* is highly aggressive on tomato and weakly aggressive on potato but is capable of causing disease when inoculums are present in high amounts (Gannibal et al., 2014). The infection of potato plants with fungi, which are mainly tomato pathogens, during crop rotation increases the inoculum of early blight and can therefore influence disease progression on the tomato crop. Early blight symptoms on the leaves include dark brown to black spots with concentric rings, which may be produced by a variety of large-spored *Alternaria* organisms. Since the disease is mainly controlled by reducing soil-borne inoculum, it is critical to pay attention to the adjacent crops.

Although *A. solani* was regarded as the most dominant species liable for potato early blight, other large-spored *Alternaria* species that showed potential give rise to disease. Different sizes and shapes of leaf spots indicate that different species are present on potato in Serbia. These results indicate that these species cannot be considered residents of potato leaves as saprophytes but take part in the spread of the disease. *Alternaria* pathogens overwinter in contaminated potato tubers and plant material (Rotem, 1994). In addition to the other members of the Solanaceae family, weed plants may also serve as reservoirs of primary inoculum to start epidemics of the disease (Rotem, 1994; Blagojević et al., 2020). Consequently, the control of early blight will be a substantial challenge in the future.

Diverse studies have investigated the population genetic structure of *Alternaria* spp. originated from potato worldwide, especially *A. solani*, which has been described as a main pathogen of this host (Van der Waals et al., 2004; Meng et al., 2015; Adhikari et al., 2020). The presence of cryptic species belonging to the *A. solani* complex, which are hard to distinguish without molecular tools, indicates that further investigation of the etiology of disease will be needed for the successful improvement of a disease management program.

## Data Availability Statement

The datasets presented in this study can be found in online repositories. The names of the repository/repositories and accession number(s) can be found in the article/Supplementary Material.

## Author Contributions

ŽI, BI, and GJ contributed to conception and design of the study. DŽ organized the database. JB performed the statistical analysis. ŽI wrote the first draft of the manuscript. JB wrote sections of the manuscript. All authors contributed to manuscript revision, read, and approved the submitted version.

## Funding

This work was supported by Ministry of education, science and technological development of Republic of Serbia, Contract no. 451–03-9/2021–14/200010.

## Conflict of Interest

The authors declare that the research was conducted in the absence of any commercial or financial relationships that could be construed as a potential conflict of interest.

## Publisher’s Note

All claims expressed in this article are solely those of the authors and do not necessarily represent those of their affiliated organizations, or those of the publisher, the editors and the reviewers. Any product that may be evaluated in this article, or claim that may be made by its manufacturer, is not guaranteed or endorsed by the publisher.

## Supplementary Material

Supplementary Table 1 Primers used in PCR assays.

Supplementary Table 2 Isolates of *Alternaria* spp. identified in this study and retrieved from the GenBank database and GenBank accession numbers for *GAPDH*, *RPB2* and *Calmodulin* genes.

Supplementary Figure 1 a) PCR-based identification that amplified a 164 bp fragment from the *Alt a1* gene with primer pair OAsF7/OAsR6 to separate specie *A. solani* and related species (*A. grandis* and *A. protenta*) from *A. linariae*; b) PCR-based identification that amplified a 483 pb fragment with primer pair OAtF4/OAtR2 from the calmodulin encoding gene of *A. linariae*; c) Typical restriction patterns obtained by double digestion of PCR product of the calmodulin gene from *A. solani/**A. protenta* and *A. grandis*. Size of the larger fragment, 420 bp for *A. solani/A*. protenta and 292 bp for *A. grandis*. DNA marker: 100-bp DNA ladder (Solis BioDyne).

Supplementary Figure 2 Phylogenetic tree based on maximum likelihood analysis of the *calmodulin* genes of *Alternaria* strains from potato and *Alternaria* reference strains from NCBI data base. Bar: the estimated nucleotide substitutions per site are 0.05. The numbers near each branch represent percentages out of 1,000 bootstrap replications.

Supplementary Figure 3 Phylogenetic tree based on maximum likelihood analysis of the *GAPDH* genes of *Alternaria* strains from potato and *Alternaria* reference strains from NCBI data base. Bar: the estimated nucleotide substitutions per site are 0.01. The numbers near each branch represent percentages out of 1,000 bootstrap replications.

Supplementary Figure 4 Phylogenetic tree based on maximum likelihood analysis of the *Rpb2* genes of *Alternaria* strains from potato and *Alternaria* reference strains from NCBI data base. Bar: the estimated nucleotide substitutions per site are 0.1. The numbers near each branch represent percentages out of 1,000 bootstrap replications.
