# Supplementary material for: New Insight in the Occurrence of Early Blight Disease on Potato Reveals High Distribution of Alternaria solani and Alternaria protenta in Serbia
Source: Front Microbiol. 2022 Mar 23;13:856898. doi: 10.3389/fmicb.2022.856898 (PMC8984275; doi:10.3389/fmicb.2022.856898)
Supplement: Supplementary Table 1 — Primers used in PCR assays. [file Table_1.DOCX]

| **Locus** | **Primer name** | **Primer sequence (5'–3')** | **Reference** |
| --- | --- | --- | --- |
| *Calmodulin* | CALDF1 | AGCAAGTCTCCGAGTTCAAGG | Lawrence et al. 2013 |
|  | CALDR1 | CTTCTGCATCATCAYCTGGACG |  |
| *RPB2* | RPB2DF | ACCGACACACAAATGCTGGAGC | Lawrence et al. 2013 |
|  | RPB2DR | CAAGACCCCAATGAGAGTTGTG |  |
| *GAPDH* | GPD1 | CAA CGG CTT CGG TCG CATTG | Berbee et al. 1999 |
|  | GPD2 | GCC AAG CAG TTG GTTGTGC |  |
| Specific primers *A. solani, A. protenta* and *A. grandis* | OAsF7 | CGACGAGTAAGTTGCCCTCA | Gannibal et al. 2014 |
|  | OAsR6 | TGTAGGCGTCAGAGACACCATT |  |
| Specific primers *A. tomatophila* and other large-spored *Alternaria* spp., excluding *A. solani, A. protenta* and *A. grandis* | OAtF4 | TGCGGCTTGCTGGCTAAGGT | Gannibal et al. 2014 |
|  | OAtR2 | CAGTCGATGCGGCCGTCA |  |
