# Supplementary material for: New Insight in the Occurrence of Early Blight Disease on Potato Reveals High Distribution of Alternaria solani and Alternaria protenta in Serbia
Source: Front Microbiol. 2022 Mar 23;13:856898. doi: 10.3389/fmicb.2022.856898 (PMC8984275; doi:10.3389/fmicb.2022.856898)
Supplement: Supplementary Table 2 — Isolates of Alternaria spp. identified in this study and retrieved from the GenBank database and GenBank accession numbers for GAPDH, RPB2 and Calmodulin genes. [file Table_2.DOC]

| Strains | Species | Disrict | Area of origin | Reference | GenBank Accession numbers | | | |
| --- | --- | --- | --- | --- | --- | --- | --- | --- |
|  |  |  |  |  | *GAPDH* | *Rpb2* | | *Calmodulin* |
| IZB 1R | *A. protenta* | Pomoravlje (Central Serbia) | Međureč | This study | MW645385 | MW645422 | MW591972 | |
| IZB 1R1 | *A. protenta* | Pomoravlje (Central Serbia) | Međureč | This study |  |  |  | |
| IZB 1R3 | *A. protenta* | Pomoravlje (Central Serbia) | Međureč | This study |  |  |  | |
| IZB 1RM | *A. protenta* | Pomoravlje (Central Serbia) | Međureč | This study |  |  |  | |
| IZB 1RM1 | *A. protenta* | Pomoravlje (Central Serbia) | Međureč | This study |  |  |  | |
| IZB 1SS | *A. protenta* | Pomoravlje (Central Serbia) | Međureč | This study | MW645386 | MW645423 | MW591973 | |
| IZB 2SS | *A. protenta* | Pomoravlje (Central Serbia) | Međureč | This study |  |  |  | |
| IZB 2R | *A. protenta* | Pomoravlje (Central Serbia) | Međureč | This study |  |  |  | |
| IZB 2R1 | *A. protenta* | Pomoravlje (Central Serbia) | Međureč | This study |  |  |  | |
| IZB 2R2 | *A. protenta* | Pomoravlje (Central Serbia) | Međureč | This study |  |  |  | |
| IZB 2S1 | *A. protenta* | Pomoravlje (Central Serbia) | Međureč | This study |  |  |  | |
| IZB 2S2 | *A. protenta* | Pomoravlje (Central Serbia) | Međureč | This study |  |  |  | |
| IZB 2S5 | *A. protenta* | Pomoravlje (Central Serbia) | Međureč | This study |  |  |  | |
| IZB 2S7 | *A. protenta* | Pomoravlje (Central Serbia) | Međureč | This study |  |  |  | |
| IZB 3R | *A. protenta* | Pomoravlje (Central Serbia) | Međureč | This study |  |  |  | |
| IZB 3R1 | *A. protenta* | Pomoravlje (Central Serbia) | Međureč | This study |  |  |  | |
| IZB 24-1 | *A. tomatophila* | Pomoravlje (Central Serbia) | Dragovcet | This study | MW645397 | MW645434 | MW591984 | |
| IZB 24-2 | *A. tomatophila* | Pomoravlje (Central Serbia) | Dragovcet | This study |  |  |  | |
| IZB 24-7 | *A. tomatophila* | Pomoravlje (Central Serbia) | Dragovcet | This study |  |  |  | |
| IZB 24-11 | *A. tomatophila* | Pomoravlje (Central Serbia) | Dragovcet | This study | MW645398 | MW645435 | MW591985 | |
| IZB 25-1 | *A. protenta* | Pomoravlje (Central Serbia) | Dragovcet | This study |  |  |  | |
| IZB 25-3 | *A. protenta* | Pomoravlje (Central Serbia) | Dragovcet | This study |  |  |  | |
| IZB 25-4 | *A. protenta* | Pomoravlje (Central Serbia) | Dragovcet | This study |  |  |  | |
| IZB 25-5 | *A. protenta* | Pomoravlje (Central Serbia) | Dragovcet | This study |  |  |  | |
| IZB 25-6 | *A. solani* | Pomoravlje (Central Serbia) | Dragovcet | This study | MW645373 | MW645410 | MW591960 | |
| IZB 25-7 | *A. protenta* | Pomoravlje (Central Serbia) | Dragovcet | This study |  |  |  | |
| IZB 25-9 | *A. protenta* | Pomoravlje (Central Serbia) | Dragovcet | This study |  |  |  | |
| IZB 36-1 | *A. protenta* | Pomoravlje (Central Serbia) | Dragovcet | This study |  |  |  | |
| IZB 36-2 | *A. protenta* | Pomoravlje (Central Serbia) | Dragovcet | This study |  |  |  | |
| IZB 36-4 | *A. solani* | Pomoravlje (Central Serbia) | Dragovcet | This study |  |  |  | |
| IZB 36k | *A. solani* | Pomoravlje (Central Serbia) | Dragovcet | This study |  |  |  | |
| IZB 36k-1 | *A. solani* | Pomoravlje (Central Serbia) | Dragovcet | This study |  |  |  | |
| IZB 1k-1 | *A. protenta* | Jablanica (Southern Serbia) | Bogojevce | This study | MW645387 | MW645424 | MW591974 | |
| IZB 1k-2 | *A. solani* | Jablanica (Southern Serbia) | Bogojevce | This study | MW645369 | MW645406 | MW591956 | |
| IZB 1k-4 | *A. solani* | Jablanica (Southern Serbia) | Bogojevce | This study |  |  |  | |
| IZB 1k-5 | *A. protenta* | Jablanica (Southern Serbia) | Bogojevce | This study |  |  |  | |
| IZB 4-1 | *A. protenta* | Jablanica (Southern Serbia) | Navalin | This study |  |  |  | |
| IZB 4-3 | *A. protenta* | Jablanica (Southern Serbia) | Navalin | This study | MW645388 | MW645425 | MW591975 | |
| IZB 6 | *A. solani* | Jablanica (Southern Serbia) | Bogojevce | This study |  |  |  | |
| IZB 6R | *A. solani* | Jablanica (Southern Serbia) | Bogojevce | This study |  |  |  | |
| IZB 6R1 | *A. solani* | Jablanica (Southern Serbia) | Bogojevce | This study |  |  |  | |
| IZB 6R2 | *A. solani* | Jablanica (Southern Serbia) | Bogojevce | This study |  |  |  | |
| IZB 6R4 | *A. solani* | Jablanica (Southern Serbia) | Bogojevce | This study |  |  |  | |
| IZB 6-1 | *A. solani* | Jablanica (Southern Serbia) | Bogojevce | This study | MW645370 | MW645407 | MW591957 | |
| IZB 6-2 | *A. solani* | Jablanica (Southern Serbia) | Bogojevce | This study |  |  |  | |
| IZB 6-3 | *A. solani* | Jablanica (Southern Serbia) | Bogojevce | This study |  |  |  | |
| IZB 6-4 | *A. solani* | Jablanica (Southern Serbia) | Bogojevce | This study |  |  |  | |
| IZB 6-5 | *A. solani* | Jablanica (Southern Serbia) | Bogojevce | This study |  |  |  | |
| IZB 6-7 | *A. solani* | Jablanica (Southern Serbia) | Bogojevce | This study |  |  |  | |
| IZB 6k-1 | *A. protenta* | Jablanica (Southern Serbia) | Bogojevce | This study |  |  |  | |
| IZB 6k-3 | *A. protenta* | Jablanica (Southern Serbia) | Bogojevce | This study |  |  |  | |
| IZB 6k-4 | *A. solani* | Jablanica (Southern Serbia) | Bogojevce | This study |  |  |  | |
| IZB 7 | *A. protenta* | Jablanica (Southern Serbia) | Navalin | This study |  |  |  | |
| IZB 7-1 | *A. protenta* | Jablanica (Southern Serbia) | Navalin | This study |  |  |  | |
| IZB 7-2 | *A. protenta* | Jablanica (Southern Serbia) | Navalin | This study |  |  |  | |
| IZB 7-3 | *A. protenta* | Jablanica (Southern Serbia) | Navalin | This study |  |  |  | |
| IZB 7-5 | *A. protenta* | Jablanica (Southern Serbia) | Navalin | This study |  |  |  | |
| IZB 7-6 | *A. protenta* | Jablanica (Southern Serbia) | Navalin | This study |  |  |  | |
| IZB 7k-1 | *A. protenta* | Jablanica (Southern Serbia) | Bogojevce | This study |  |  |  | |
| IZB 7k-2 | *A. protenta* | Jablanica (Southern Serbia) | Bogojevce | This study |  |  |  | |
| IZB 7k-3 | *A. solani* | Jablanica (Southern Serbia) | Bogojevce | This study |  |  |  | |
| IZB 7k-5 | *A. solani* | Jablanica (Southern Serbia) | Bogojevce | This study |  |  |  | |
| IZB 7k-6 | *A. solani* | Jablanica (Southern Serbia) | Bogojevce | This study |  |  |  | |
| IZB 7k-7 | *A. solani* | Jablanica (Southern Serbia) | Bogojevce | This study |  |  |  | |
| IZB 7k-8 | *A. solani* | Jablanica (Southern Serbia) | Bogojevce | This study |  |  |  | |
| IZB 7k-10 | *A. solani* | Jablanica (Southern Serbia) | Bogojevce | This study |  |  |  | |
| IZB 7k-11 | *A. protenta* | Jablanica (Southern Serbia) | Bogojevce | This study |  |  |  | |
| IZB 7k-12 | *A. protenta* | Jablanica (Southern Serbia) | Bogojevce | This study |  |  |  | |
| IZB 9k-2 | *A. protenta* | Jablanica (Southern Serbia) | Pečenjevce | This study |  |  |  | |
| IZB 9k-3 | *A. protenta* | Jablanica (Southern Serbia) | Pečenjevce | This study |  |  |  | |
| IZB 10k-1 | *A. protenta* | Jablanica (Southern Serbia) | Pečenjevce | This study | MW645389 | MW645426 | MW591976 | |
| IZB 10k-3 | *A. protenta* | Jablanica (Southern Serbia) | Pečenjevce | This study |  |  |  | |
| IZB 10k-4 | *A. protenta* | Jablanica (Southern Serbia) | Pečenjevce | This study |  |  |  | |
| IZB 10k-6 | *A. protenta* | Jablanica (Southern Serbia) | Pečenjevce | This study |  |  |  | |
| IZB 10k-8 | *A. protenta* | Jablanica (Southern Serbia) | Pečenjevce | This study |  |  |  | |
| IZB 10k-9 | *A. protenta* | Jablanica (Southern Serbia) | Pečenjevce | This study |  |  |  | |
| IZB 11k-1 | *A. solani* | Jablanica (Southern Serbia) | Brejanovce | This study | MW645371 | MW645408 | MW591958 | |
| IZB 11k-2 | *A. solani* | Jablanica (Southern Serbia) | Brejanovce | This study |  |  |  | |
| IZB 12 | *A. solani* | Jablanica (Southern Serbia) | Brejanovce | This study |  |  |  | |
| IZB 13 | *A. solani* | Jablanica (Southern Serbia) | Brejanovce | This study |  |  |  | |
| IZB 15 | *A. protenta* | Jablanica (Southern Serbia) | Brejanovce | This study |  |  |  | |
| IZB 19 | *A. protenta* | Jablanica (Southern Serbia) | Brejanovce | This study | MW645390 | MW645427 | MW591977 | |
| IZB 91A | *A. solani* | Jablanica (Southern Serbia) | Bogojevce | This study |  |  |  | |
| IZB 91A1 | *A. solani* | Jablanica (Southern Serbia) | Bogojevce | This study |  |  |  | |
| IZB 92A | *A. solani* | Jablanica (Southern Serbia) | Bogojevce | This study |  |  |  | |
| IZB 93A | *A. solani* | Jablanica (Southern Serbia) | Bogojevce | This study |  |  |  | |
| IZB 94A | *A. solani* | Jablanica (Southern Serbia) | Brejanovce | This study |  |  |  | |
| IZB 95A | *A. solani* | Jablanica (Southern Serbia) | Brejanovce | This study |  |  |  | |
| IZB 11-16 | *A. protenta* | South Bačka (Northern Serbia) | Maglić | This study |  |  |  | |
| IZB 11-17 | *A. protenta* | South Bačka (Northern Serbia) | Maglić | This study |  |  |  | |
| IZB 38 | *A. solani* | South Bačka (Northern Serbia) | Kulpin | This study |  |  |  | |
| IZB 38-1 | *A. solani* | South Bačka (Northern Serbia) | Kulpin | This study | MW645375 | MW645412 | MW591962 | |
| IZB 38-2 | *A. solani* | South Bačka (Northern Serbia) | Kulpin | This study |  |  |  | |
| IZB 38-4 | *A. solani* | South Bačka (Northern Serbia) | Kulpin | This study |  |  |  | |
| IZB 38-5 | *A. solani* | South Bačka (Northern Serbia) | Kulpin | This study |  |  |  | |
| IZB 39 | *A. solani* | South Bačka (Northern Serbia) | Kulpin | This study |  |  |  | |
| IZB 40 | *A. solani* | South Bačka (Northern Serbia) | Kulpin | This study |  |  |  | |
| IZB 41 | *A. solani* | South Bačka (Northern Serbia) | Kulpin | This study |  |  |  | |
| IZB 42 | *A. solani* | South Bačka (Northern Serbia) | Kulpin | This study |  |  |  | |
| IZB 44 | *A. solani* | South Bačka (Northern Serbia) | Kulpin | This study |  |  |  | |
| IZB 45 | *A. solani* | South Bačka (Northern Serbia) | Maglić | This study |  |  |  | |
| IZB 45-1 | *A. solani* | South Bačka (Northern Serbia) | Maglić | This study |  |  |  | |
| IZB 45-2 | *A. solani* | South Bačka (Northern Serbia) | Maglić | This study | MW645376 | MW645413 | MW591963 | |
| IZB 45-3 | *A. solani* | South Bačka (Northern Serbia) | Maglić | This study |  |  |  | |
| IZB 45-4 | *A. solani* | South Bačka (Northern Serbia) | Maglić | This study |  |  |  | |
| IZB 45-5 | *A. solani* | South Bačka (Northern Serbia) | Maglić | This study |  |  |  | |
| IZB 45-6 | *A. solani* | South Bačka (Northern Serbia) | Maglić | This study |  |  |  | |
| IZB 45-7 | *A. solani* | South Bačka (Northern Serbia) | Maglić | This study |  |  |  | |
| IZB 45-8 | *A. solani* | South Bačka (Northern Serbia) | Maglić | This study |  |  |  | |
| IZB 45-9 | *A. solani* | South Bačka (Northern Serbia) | Maglić | This study |  |  |  | |
| IZB 45-10 | *A. solani* | South Bačka (Northern Serbia) | Maglić | This study |  |  |  | |
| IZB 45-11 | *A. solani* | South Bačka (Northern Serbia) | Maglić | This study |  |  |  | |
| IZB 45-12 | *A. solani* | South Bačka (Northern Serbia) | Maglić | This study |  |  |  | |
| IZB 45-13 | *A. solani* | South Bačka (Northern Serbia) | Maglić | This study |  |  |  | |
| IZB 45-14 | *A. protenta* | South Bačka (Northern Serbia) | Maglić | This study | MW645392 | MW645429 | MW591979 | |
| IZB 45-15 | *A. solani* | South Bačka (Northern Serbia) | Maglić | This study |  |  |  | |
| IZB 45-16 | *A. protenta* | South Bačka (Northern Serbia) | Maglić | This study |  |  |  | |
| IZB 45-17 | *A. solani* | South Bačka (Northern Serbia) | Maglić | This study |  |  |  | |
| IZB 46 | *A. protenta* | South Bačka (Northern Serbia) | Maglić | This study |  |  |  | |
| IZB 46-1 | *A. solani* | South Bačka (Northern Serbia) | Maglić | This study |  |  |  | |
| IZB 46-2 | *A. solani* | South Bačka (Northern Serbia) | Maglić | This study |  |  |  | |
| IZB 46-3 | *A. solani* | South Bačka (Northern Serbia) | Maglić | This study |  |  |  | |
| IZB 46-4 | *A. protenta* | South Bačka (Northern Serbia) | Maglić | This study |  |  |  | |
| IZB 46-5 | *A. solani* | South Bačka (Northern Serbia) | Maglić | This study |  |  |  | |
| IZB 46-6 | *A. solani* | South Bačka (Northern Serbia) | Maglić | This study |  |  |  | |
| IZB 46-7 | *A. solani* | South Bačka (Northern Serbia) | Maglić | This study |  |  |  | |
| IZB 46-8 | *A. solani* | South Bačka (Northern Serbia) | Maglić | This study |  |  |  | |
| IZB 46-9 | *A. solani* | South Bačka (Northern Serbia) | Maglić | This study |  |  |  | |
| IZB 46-10 | *A. solani* | South Bačka (Northern Serbia) | Maglić | This study |  |  |  | |
| IZB 46-11 | *A. solani* | South Bačka (Northern Serbia) | Maglić | This study |  |  |  | |
| IZB 46-12 | *A. solani* | South Bačka (Northern Serbia) | Maglić | This study |  |  |  | |
| IZB 46-13 | *A. solani* | South Bačka (Northern Serbia) | Maglić | This study |  |  |  | |
| IZB 46-14 | *A. solani* | South Bačka (Northern Serbia) | Maglić | This study |  |  |  | |
| IZB 46-15 | *A. solani* | South Bačka (Northern Serbia) | Maglić | This study |  |  |  | |
| IZB 47-1 | *A. solani* | South Bačka (Northern Serbia) | Maglić | This study |  |  |  | |
| IZB 47-2 | *A. solani* | South Bačka (Northern Serbia) | Maglić | This study |  |  |  | |
| IZB 47-3 | *A. solani* | South Bačka (Northern Serbia) | Maglić | This study |  |  |  | |
| IZB 47-4 | *A. solani* | South Bačka (Northern Serbia) | Maglić | This study |  |  |  | |
| IZB 47-5 | *A. solani* | South Bačka (Northern Serbia) | Maglić | This study |  |  |  | |
| IZB 47-6 | *A. solani* | South Bačka (Northern Serbia) | Maglić | This study |  |  |  | |
| IZB 47-7 | *A. solani* | South Bačka (Northern Serbia) | Maglić | This study |  |  |  | |
| IZB 47-8 | *A. solani* | South Bačka (Northern Serbia) | Maglić | This study |  |  |  | |
| IZB 47-9 | *A. solani* | South Bačka (Northern Serbia) | Maglić | This study |  |  |  | |
| IZB 47-10 | *A. solani* | South Bačka (Northern Serbia) | Maglić | This study |  |  |  | |
| IZB 47-11 | *A. solani* | South Bačka (Northern Serbia) | Maglić | This study |  |  |  | |
| IZB 47-12 | *A. solani* | South Bačka (Northern Serbia) | Maglić | This study |  |  |  | |
| IZB 47-13 | *A. solani* | South Bačka (Northern Serbia) | Maglić | This study | MW645377 | MW645414 | MW591964 | |
| IZB 47-14 | *A. protenta* | South Bačka (Northern Serbia) | Maglić | This study | MW645393 | MW645430 | MW591980 | |
| IZB 47-15 | *A. solani* | South Bačka (Northern Serbia) | Maglić | This study |  |  |  | |
| IZB 47-16 | *A. solani* | South Bačka (Northern Serbia) | Maglić | This study |  |  |  | |
| IZB 47-17 | *A. solani* | South Bačka (Northern Serbia) | Maglić | This study |  |  |  | |
| IZB 48 | *A. protenta* | South Bačka (Northern Serbia) | Maglić | This study |  |  |  | |
| IZB48-1 | *A. solani* | South Bačka (Northern Serbia) | Maglić | This study |  |  |  | |
| IZB 49 | *A. solani* | South Bačka (Northern Serbia) | Maglić | This study |  |  |  | |
| IZB 49-1 | *A. solani* | South Bačka (Northern Serbia) | Maglić | This study |  |  |  | |
| IZB 49-2 | *A. solani* | South Bačka (Northern Serbia) | Maglić | This study |  |  |  | |
| IZB 22-2I | *A. protenta* | Moravica (Western Serbia) | Baluga | This study | MW645391 | MW645428 | MW591978 | |
| IZB 22-2II | *A. solani* | Moravica (Western Serbia) | Baluga | This study | MW645372 | MW645409 | MW591959 | |
| IZB 22-3 | *A. solani* | Moravica (Western Serbia) | Baluga | This study |  |  |  | |
| IZB 22-4 | *A. protenta* | Moravica (Western Serbia) | Baluga | This study |  |  |  | |
| IZB 22-5 | *A. protenta* | Moravica (Western Serbia) | Baluga | This study |  |  |  | |
| IZB 22-7 | *A. protenta* | Moravica (Western Serbia) | Baluga | This study |  |  |  | |
| IZB 23 | *A. protenta* | Moravica (Western Serbia) | Baluga | This study |  |  |  | |
| IZB 23-2 | *A. protenta* | Moravica (Western Serbia) | Baluga | This study |  |  |  | |
| IZB 102A | *A. solani* | Moravica (Western Serbia) | Baluga | This study |  |  |  | |
| IZB 103A | *A. solani* | Moravica (Western Serbia) | Baluga | This study |  |  |  | |
| IZB 60 | *A. solani* | Moravica (Western Serbia) | Katići | This study |  |  |  | |
| IZB 60-1 | *A. solani* | Moravica (Western Serbia) | Katići | This study | MW645379 | MW645416 | MW591966 | |
| IZB 60-2 | *A. solani* | Moravica (Western Serbia) | Katići | This study |  |  |  | |
| IZB 60-3 | *A. solani* | Moravica (Western Serbia) | Katići | This study |  |  |  | |
| IZB 60-7 | *A. solani* | Moravica (Western Serbia) | Katići | This study |  |  |  | |
| IZB 60-9 | *A. solani* | Moravica (Western Serbia) | Katići | This study |  |  |  | |
| IZB 25k | *A. solani* | Moravica (Western Serbia) | Zablaće | This study | MW645374 | MW645411 | MW591961 | |
| IZB 25k-1 | *A. solani* | Moravica (Western Serbia) | Zablaće | This study |  |  |  | |
| IZB 101A | *A. grandis* | Moravica (Western Serbia) | Zablaće | This study | MW645402 | MW645439 | MW591989 | |
| IZB102A | *A. grandis* | Moravica (Western Serbia) | Zablaće | This study |  |  |  | |
| IZB 107A | *A. grandis* | Moravica (Western Serbia) | Krivača | This study | MW645403 | MW645440 | MW591990 | |
| IZB 107-1A | *A. grandis* | Moravica (Western Serbia) | Krivača | This study |  |  |  | |
| IZB 107-2A | *A. grandis* | Moravica (Western Serbia) | Krivača | This study | MW645404 | MW645441 | MW591991 | |
| IZB 107-3A | *A. grandis* | Moravica (Western Serbia) | Krivača | This study | MW645405 | MW645442 | MW591992 | |
| IZB 107-5A | *A. grandis* | Moravica (Western Serbia) | Krivača | This study |  |  |  | |
| IZB 107-6A | *A. grandis* | Moravica (Western Serbia) | Krivača | This study |  |  |  | |
| IZB 54 | *A. protenta* | Moravica (Western Serbia) | Veles | This study |  |  |  | |
| IZB 54-1 | *A. solani* | Moravica (Western Serbia) | Veles | This study |  |  |  | |
| IZB 54-2 | *A. solani* | Moravica (Western Serbia) | Veles | This study |  |  |  | |
| IZB 54-2-1 | *A. solani* | Moravica (Western Serbia) | Veles | This study |  |  |  | |
| IZB 54-3 | *A. protenta* | Moravica (Western Serbia) | Veles | This study | MW645394 | MW645431 | MW591981 | |
| IZB 54-3-1 | *A. solani* | Moravica (Western Serbia) | Veles | This study |  |  |  | |
| IZB 54-4 | *A. solani* | Moravica (Western Serbia) | Veles | This study |  |  |  | |
| IZB 54-5-1 | *A. solani* | Moravica (Western Serbia) | Veles | This study |  |  |  | |
| IZB 54-6 | *A. solani* | Moravica (Western Serbia) | Veles | This study |  |  |  | |
| IZB 54-7 | *A. solani* | Moravica (Western Serbia) | Veles | This study | MW645378 | MW645415 | MW591965 | |
| IZB 54-8 | *A. protenta* | Moravica (Western Serbia) | Veles | This study |  |  |  | |
| IZB 54-9 | *A. protenta* | Moravica (Western Serbia) | Veles | This study |  |  |  | |
| IZB 54-10 | *A. solani* | Moravica (Western Serbia) | Veles | This study |  |  |  | |
| IZB 54-11 | *A. grandis* | Moravica (Western Serbia) | Veles | This study | MW645401 | MW645438 | MW591988 | |
| IZB 54-12 | *A. solani* | Moravica (Western Serbia) | Veles | This study |  |  |  | |
| IZB 54-13 | *A. grandis* | Moravica (Western Serbia) | Veles | This study |  |  |  | |
| IZB 54-15 | *A. solani* | Moravica (Western Serbia) | Veles | This study |  |  |  | |
| IZB 54-17 | *A. solani* | Moravica (Western Serbia) | Veles | This study |  |  |  | |
| IZB 54-18 | *A. solani* | Moravica (Western Serbia) | Veles | This study |  |  |  | |
| IZB 54-18-1 | *A. protenta* | Moravica (Western Serbia) | Veles | This study |  |  |  | |
| IZB 54-18-2 | *A. protenta* | Moravica (Western Serbia) | Veles | This study |  |  |  | |
| IZB 54-19 | *A. solani* | Moravica (Western Serbia) | Veles | This study |  |  |  | |
| IZB 54-19-1 | *A. solani* | Moravica (Western Serbia) | Veles | This study |  |  |  | |
| IZB 54-20 | *A. solani* | Moravica (Western Serbia) | Veles | This study |  |  |  | |
| IZB 54-21 | *A. solani* | Moravica (Western Serbia) | Veles | This study |  |  |  | |
| IZB 54-22 | *A. solani* | Moravica (Western Serbia) | Veles | This study |  |  |  | |
| IZB 36t | *A. tomatophila* | Belgrade (Northern Serbia) | Borča | This study |  |  |  | |
| IZB 36c-5 | *A. tomatophila* | Belgrade (Northern Serbia) | Borča | This study | MW645399 | MW645436 | MW591986 | |
| IZB 61 | *A. solani* | Zlatibor (Western Serbia) | Drmanovići | This study | MW645379 | MW645417 | MW591967 | |
| IZB 61-2 | *A. solani* | Zlatibor (Western Serbia) | Drmanovići | This study |  |  |  | |
| IZB 62 | *A. solani* | Zlatibor (Western Serbia) | Kladnica | This study | MW645381 | MW645418 | MW591968 | |
| IZB62-2 | *A. solani* | Zlatibor (Western Serbia) | Kladnica | This study |  |  |  | |
| IZB 62-3 | *A. solani* | Zlatibor (Western Serbia) | Kladnica | This study |  |  |  | |
| IZB 62-5 | *A. solani* | Zlatibor (Western Serbia) | Kladnica | This study |  |  |  | |
| IZB 81A | *A. protenta* | North Bačka (Northern Serbia) | Zobnatica | This study |  |  |  | |
| IZB 82A | *A. solani* | North Bačka (Northern Serbia) | Zobnatica | This study |  |  |  | |
| IZB 83A | *A. protenta* | North Bačka (Northern Serbia) | Zobnatica | This study | MW645395 | MW645432 | MW591982 | |
| IZB 83Aprs | *A. solani* | North Bačka (Northern Serbia) | Zobnatica | This study | MW645382 | MW645419 | MW591969 | |
| IZB 84A | *A. solani* | North Bačka (Northern Serbia) | Zobnatica | This study |  |  |  | |
| IZB 85A1 | *A. solani* | North Bačka (Northern Serbia) | Zobnatica | This study | MW645383 | MW645420 | MW591970 | |
| IZB 87A | *A. solani* | North Bačka (Northern Serbia) | Zobnatica | This study |  |  |  | |
| IZB 88A | *A. solani* | North Bačka (Northern Serbia) | Zobnatica | This study |  |  |  | |
| IZB 120A | *A. protenta* | Raška (Western Serbia) | Bzovik | This study |  |  |  | |
| IZB 121A | *A. protenta* | Raška (Western Serbia) | Bzovik | This study |  |  |  | |
| IZB 124A | *A. protenta* | Raška (Western Serbia) | Bzovik | This study | MW645396 | MW645433 | MW591983 | |
| IZB 125A | *A. protenta* | Raška (Western Serbia) | Bzovik | This study |  |  |  | |
| IZB 128A | *A. solani* | Raška (Western Serbia) | Rudno | This study | MW645384 | MW645421 | MW591971 | |
| IZB 129A | *A. solani* | Raška (Western Serbia) | Rudno | This study |  |  |  | |
| IZB 135A | *A. tomatophila* | Rasina (Central Serbia) | Velika Drenova | This study |  |  |  | |
| IZB 136A | *A. tomatophila* | Rasina (Central Serbia) | Velika Drenova | This study | MW645400 | MW645436 | MW591987 | |
| NB251 | *A. grandis* | - | Algeria | Bassadat et al 2016 |  |  | KR911766 | |
| DA009 | *A. grandis* | - | Algeria | Ayad et al 2017 |  |  | MH243790 | |
| DA052 | *A. grandis* | - | Algeria | Ayad et al 2017 |  |  | MH243773 | |
| CBS 109158 | *A. grandis* | - | USA | Lawrence et al 2013 | JQ646341 | JQ646500 | JQ646249, | |
| DA119 | *A. linariae* | - | Algeria | Ayad et al 2017 |  |  | MH243800 | |
| DA100 | *A. linariae* | - | Algeria | Ayad et al 2017 |  |  | MH243807 | |
| MF-P138061 | *A. tomatophila* | - | Russia | Gannibal et al 2012 | KJ397985 |  | KJ397980 | |
| CBS 109156 | *A. tomatophila* | - | USA | Lawrence et al 2013 | JQ646347 | JQ646485 | JQ646257 | |
| CBS 116696 | *A. protenta* | - | Israel | Lawrence et al 2013 | JQ646335 | KJ718394 | JQ646236 | |
| CBS 109157 (EGS 44-098) | *A. solani* | - | USA | Woudenberg et al 2014 | GQ180080 | KJ718413 | KJ397981 | |
| MF-P048011 | *A. solani* | - | Russia | Gannibal et al 2012 | KJ397984 |  | KJ397979 | |
| BMP 0384 | *E. pedicellatum* | - | USA | Lawrence et al 2013 | AY278824 | JQ646512 | JQ646099 | |
