# Supplementary figures and images for: New Insight in the Occurrence of Early Blight Disease on Potato Reveals High Distribution of Alternaria solani and Alternaria protenta in Serbia
Source: Front Microbiol. 2022 Mar 23;13:856898. doi: 10.3389/fmicb.2022.856898 (PMC8984275; doi:10.3389/fmicb.2022.856898)

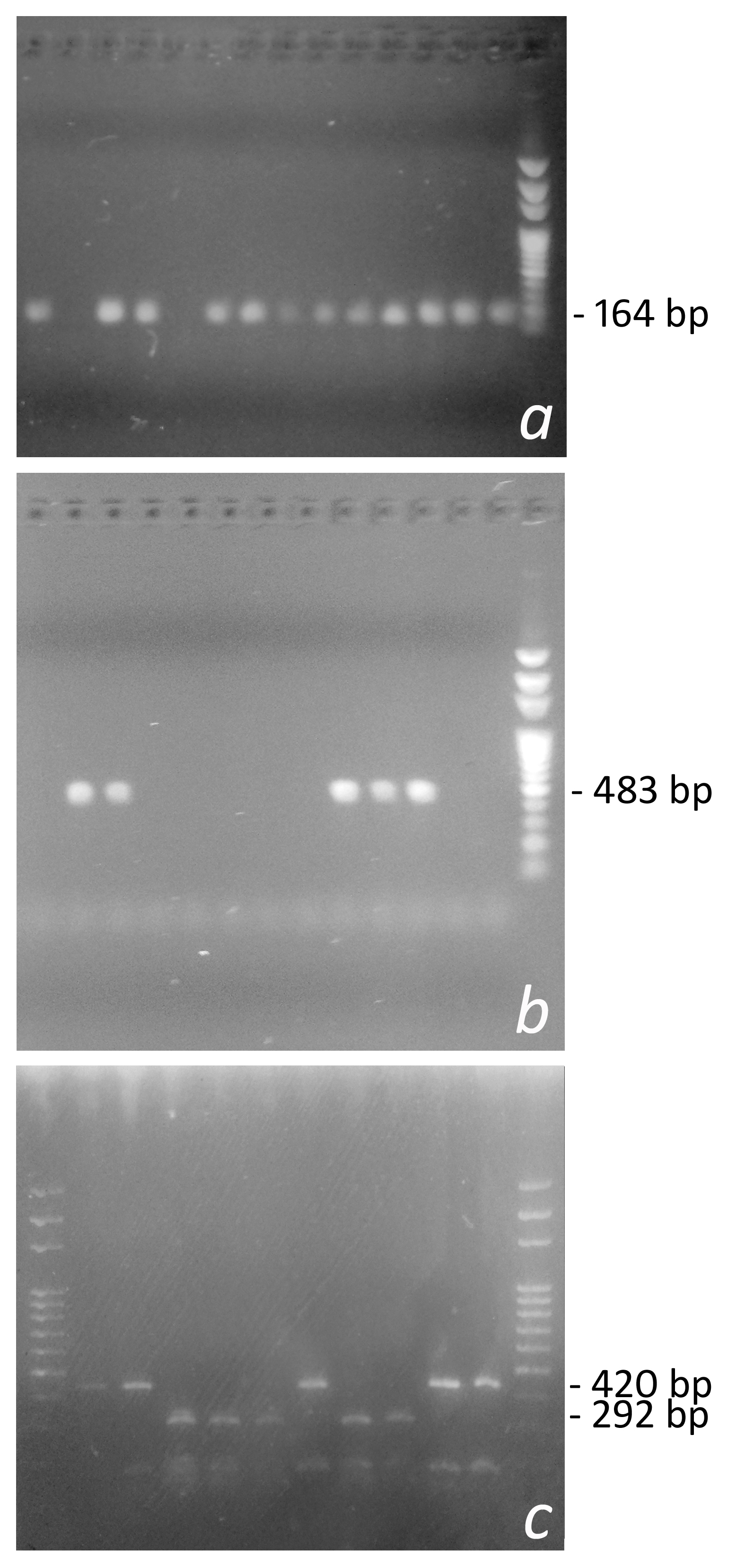

Supplement: Supplementary Figure 1 — a) PCR-based identification that amplified a 164 bp fragment from the Alt a1 gene with primer pair OAsF7/OAsR6 to separate specie A. solani and related species (A. grandis and A. protenta) from A. linariae; b) PCR-based identification that amplified a 483 pb fragment with primer pair OAtF4/OAtR2 from the calmodulin encoding gene of A. linariae; c) Typical restriction patterns obtained by double digestion of PCR product of the calmodulin gene from A. solani/A. protenta and A. grandis. Size of the larger fragment, 420 bp for A. solani/A. protenta and 292 bp for A. grandis. DNA marker: 100-bp DNA ladder (Solis BioDyne). [file Image_1.TIF]

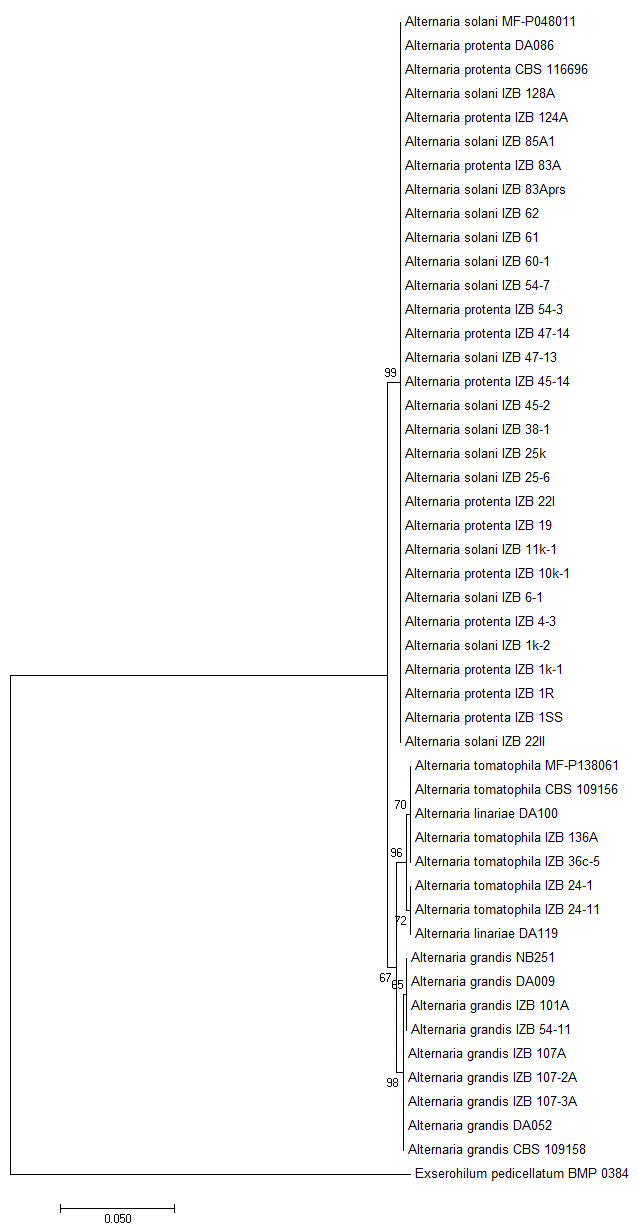

Supplement: Supplementary Figure 2 — Phylogenetic tree based on maximum likelihood analysis of the calmodulin genes of Alternaria strains from potato and Alternaria reference strains from NCBI data base. Bar: the estimated nucleotide substitutions per site are 0.05. The numbers near each branch represent percentages out of 1,000 bootstrap replications. [file Image_2.TIF]

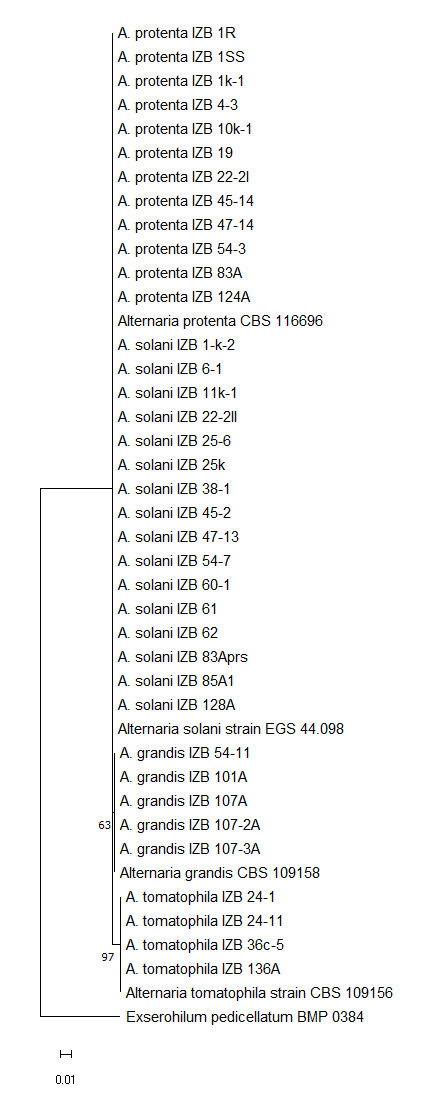

Supplement: Supplementary Figure 3 — Phylogenetic tree based on maximum likelihood analysis of the GAPDH genes of Alternaria strains from potato and Alternaria reference strains from NCBI data base. Bar: the estimated nucleotide substitutions per site are 0.01. The numbers near each branch represent percentages out of 1,000 bootstrap replications. [file Image_3.TIF]

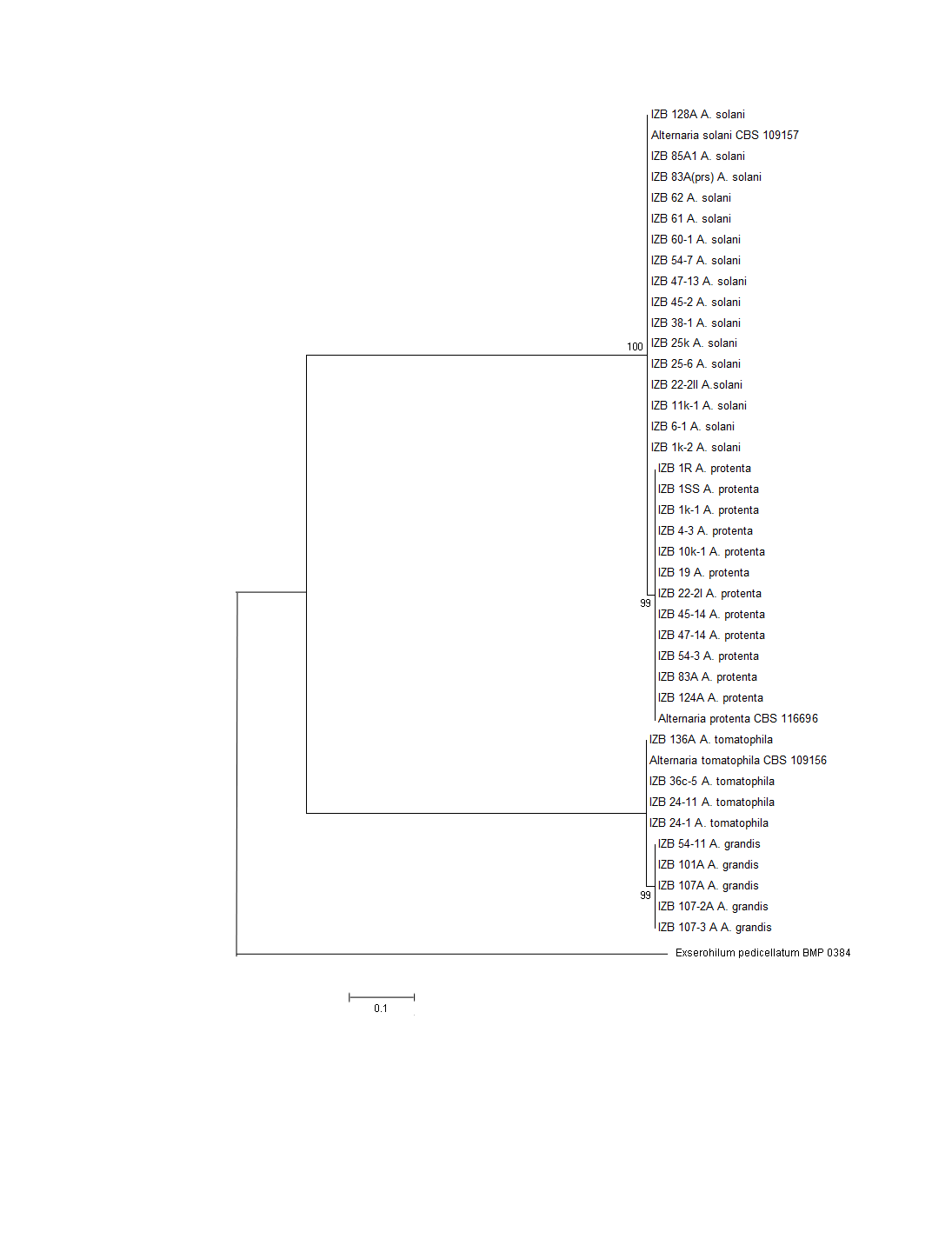

Supplement: Supplementary Figure 4 — Phylogenetic tree based on maximum likelihood analysis of the Rpb2 genes of Alternaria strains from potato and Alternaria reference strains from NCBI data base. Bar: the estimated nucleotide substitutions per site are 0.1. The numbers near each branch represent percentages out of 1,000 bootstrap replications. [file Image_4.TIF]
